# Stem Cells for Cutaneous Wound Healing

**DOI:** 10.1155/2015/285869

**Published:** 2015-06-02

**Authors:** Giles T. S. Kirby, Stuart J. Mills, Allison J. Cowin, Louise E. Smith

**Affiliations:** ^1^Mawson Institute, University of South Australia, Mawson Lakes, Adelaide, SA 5095, Australia; ^2^School of Engineering, University of South Australia, Mawson Lakes, Adelaide, SA 5095, Australia

## Abstract

Optimum healing of a cutaneous wound involves a well-orchestrated cascade of biological and molecular processes involving cell migration, proliferation, extracellular matrix deposition, and remodelling. When the normal biological process fails for any reason, this healing process can stall resulting in chronic wounds. Wounds are a growing clinical burden on healthcare systems and with an aging population as well as increasing incidences of obesity and diabetes, this problem is set to increase. Cell therapies may be the solution. A range of cell based approaches have begun to cross the rift from bench to bedside and the supporting data suggests that the appropriate administration of stem cells can accelerate wound healing. This review examines the main cell types explored for cutaneous wound healing with a focus on clinical use. The literature overwhelmingly suggests that cell therapies can help to heal cutaneous wounds when used appropriately but we are at risk of clinical use outpacing the evidence. There is a need, now more than ever, for standardised methods of cell characterisation and delivery, as well as randomised clinical trials.

## 1. Introduction

Skin is the largest organ in the human body and features a range of complex structures. The main function of the skin is to act as a barrier. Skin is formed of two distinct tissues: the epidermis and dermis. The epidermis is the outermost covering and provides protection from water and pathogens. This layer is mainly composed of keratinocytes although it also contains melanocytes, Langerhans cells, and Merkel cells [1]. The dermis is situated below the epidermis and consists of connective tissue populated with fibroblasts. The dermis provides cushioning and tensile strength to the skin through an extracellular matrix consisting of collagen fibre bundles in a basket weave arrangement, all embedded within proteoglycans [2].

Chronic wounds are rarely seen in otherwise healthy individuals; they are often associated with diabetes or obesity. It has been estimated that 1-2% of people in developed countries will suffer from chronic wounds in their lifetime [3] and in Scandinavian countries the associated costs for chronic wounds account for 2–4% of total healthcare expenses [4]. With an aging population and increasing rates of obesity and diabetes, it is clear that this problem is set to increase. Healthcare systems are in desperate need of alternative therapies and stem cells may well be the answer. With the clinical need set to grow we are now, more than ever, in need of innovative solutions if we hope to keep healthcare budgets under control.

**Figure 1:**
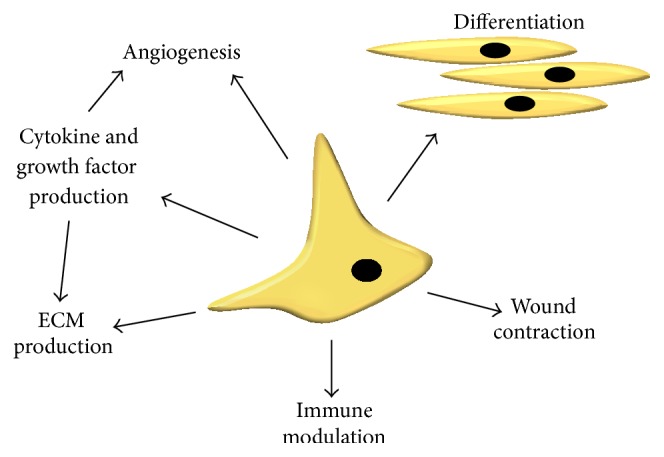
Proposed modes of action of a cell therapy.

Normal wound healing is a complex and well-orchestrated process consisting of inflammation, matrix formation, and remodelling. Cell therapies offer a huge potential in the field of cutaneous wound healing and are thought to act in a number of ways to assist in wound repair (Figure 1). This combined mode of action is why cell therapies are thought to be more effective than a simpler alternative such as direct growth factor therapy treatment. Furthermore, a limitation of direct cytokine and growth factor treatment is the inherently low stability and short* in vivo *half-life for growth factors [5, 6] as well as the potential risks involved with the delivery of a single growth factor. For example, the delivery of platelet derived growth factor (PDGF) can increase incidences of cancer mortality [7]. By using live cells we can negate a host of delivery limitations leading to better therapies.

Current methods of wound management are palliative, but their ineffectiveness for complex wounds is an ongoing clinical problem. There are a growing number of studies demonstrating the effectiveness of cell therapies in the repair/regeneration of cutaneous wounds (Table 1). This is only part of the story; hurdles still need to be overcome and these need to be commercially driven. Safety, regulatory hurdles, and cost are key obstacles for the successful uptake of clinical therapies. Fortunately, the gaps between academia and industry are closing with the successful implementation of worldwide academic/industrial/government collaborations and endeavours. The last decade has seen a focus on bench-to-bedside approaches to tackle this translational rift.

Cell therapy is most appropriately defined as a set of strategies to use living cells as a regenerative therapy to repair, replace, or restore biological function. Stem cells are thought to have influence during normal repair/regeneration; thus a large proportion of research focuses on stem cells. Adult stem cells are the most common as they are relatively easy to obtain and culture. Importantly, this cell source does not raise the same ethical concerns as embryonic stem cells. Adult stem cells can be obtained from almost any tissue but bone marrow, blood, and adipose derived cells are the most widely reported in the literature. A potential risk of adult stem cells is the potential for disease transmission but this risk can be mitigated with strict screening and exclusion criteria for cell donors. Autologous therapies may negate disease risks but a bigger concern is that of malignancy development. There have been clinical reports of carcinoma following autograft treatment [8]. It is hoped that further research will result in safer and more effective cell therapies.

This review assesses the current state of human stem cell therapies for cutaneous wounds with a particular focus on cell types, clinical trials, and current limitations.

## 2. Cutaneous Wound Repair

The purpose of wound healing is to repair the skin to prevent infection and to restore tissue integrity and function. Unfortunately, in adults, this process is geared towards faster rates of healing, to prevent infection, which ultimately leads to a compromise in the quality of healing. This compromise results in the formation of a scar, where the architecture of the skin is distinct from the original tissue and is often accompanied by a loss of function and pain which can also have an impact psychologically. The ideal for future treatments is to increase the rates of healing whilst improving the quality of healing resulting in more of a regenerative process rather than a repair orientated one. The wound healing process can be considered to consist of three overlapping phases, which are inflammation, proliferation, and remodelling, where a disruption to any of these phases can result in delayed or even incomplete healing.

### 2.1. Initial Insult and Inflammation

Wound healing is initiated after an insult to the skin, which is often accompanied by a breach in vessel wall integrity leading to the extravasation of blood. This causes the platelets to aggregate and degranulate, which results in the activation of the coagulation cascade and the formation of a fibrin clot, preventing further blood loss [9–12]. The inflammatory process begins when immune cells are attracted to the wound site by a milieu of cytokines, growth factors, and cellular debris as well as invading pathogens such as bacteria, viruses, and fungi [13]. The first immune cells to migrate to the wound site are neutrophils in response to PDGF and transforming growth factor-beta (TGF-*β*) [14, 15]. Soon after, macrophages are then attracted to the wound site in response to cytokines and growth factors released by the neutrophils [16]. Both macrophages and neutrophils can identify invading pathogens and damaged tissue via their toll-like receptors (TLRs) and upon activation of these receptors there is an increase in the expression of proinflammatory cytokines [17], which leads to more immune cells being recruited to the wound site. Together both of these cell types clear the wound of debris and infection via phagocytosis [16]. Macrophages then play a role in terminating the inflammatory process by expressing anti-inflammatory cytokines such as vascular endothelial growth factor (VEGF) and interleukin-10 (IL-10). Inflammation is a key process which can determine not only the rate of healing but also the degree of fibrosis as excessive inflammatory reactions have shown to result in delayed or incomplete healing and an increase in the severity of fibrosis [18, 19]. Reepithelialisation also begins at this time with an increase in expression of matrix metalloproteinases (MMPs) and plasmin by the wound edge keratinocytes to degrade the fibrin clot [20, 21]. This creates a space for the keratinocytes to proliferate and migrate beneath the clot. Once the epithelium is reestablished the basement membrane is reformed.

### 2.2. Matrix Formation

Matrix formation begins around 72 hours after wounding and is facilitated by fibroblasts. Fibroblasts enter the wound site in response to growth factors including PDGF and TGF-*β* [22]. Once within the wound the fibroblasts initially deposit collagen III, fibronectin, and hyaluronan. Angiogenesis, the process of reforming blood vessels throughout the injured skin, also occurs around this phase. A blood supply is required to supply the injured skin with nutrients and oxygen to enable cellular migration, proliferation, and differentiation. This process is initiated by the release of VEGF and fibroblast growth factor-2 (FGF-2) from damaged endothelial cells, keratinocytes, and macrophages [23]. This enables the endothelial cells to proliferate and migrate into the wound site to form a new blood vessel network. This action also requires the proteolysis and reformation of the dermal matrix similar to reepithelialisation.

### 2.3. Remodelling

This phase includes events such as collagen synthesis, degradation and reorganisation, and often the formation of scar tissue. There is a replacement of fibronectin and hyaluronan by heparin sulphate in the basement membrane and dermatan and chondroitin sulphate in the interstitium [9]. There is also the gradual replacement of collagen III with collagen I. This process is tightly controlled and regulated by the expression MMPs and tissue inhibitors of MMPs (TIMPs) [24]. MMPs are responsible for the degradation of the collagen network whilst the TIMPs act, by direct 1 : 1 binding of the appropriate MMPs, to inhibit their action [25]. Ideally, the remodelling of the dermal matrix would reform an exact replica of the original skin, which can be observed in the healing of embryos up until the third trimester of gestation [26, 27]. In adult healing however this process is often flawed, in preference for accelerated healing, with the new tissue being architecturally distinct from the original and this can result in scar formation [28], which can lead to a loss of function in the newly formed skin as well as having a psychological impact and the occurrence of pain. Scar tissue consists of fibrous collagen but lacks the random basket weave architecture of normal tissue; the collagen fibres tend to be more aligned. In addition, scar tissue lacks a range of physiological epidermal appendages such as follicles or sweat glands.

## 3. Mesenchymal Stem Cells

Mesenchymal stem cells (MSCs) are a multipotent cell type that arises from the embryonic connective tissue—mesenchyme. Their multipotent properties enable them to readily differentiate into several different cell types including osteoblasts, chondrocytes, adipocytes, tenocytes, and myocytes, under specific culture conditions [29, 30]. Bone marrow is the prominent source but MSCs have in fact been found in various niches throughout the body. These include Wharton's jelly, the umbilical cord blood, adipose tissue, bone marrow, dental pulp, muscle, and skin [31]. It has been suggested that the term MSCs should be redefined to “multipotent mesenchymal stromal cells” in order to cover the increasing range of MSC niches utilised [32]. In 2006 the Mesenchymal and Tissue Stem Cell Committee of the International Society for Cellular Therapy proposed a set of standard characteristics for stem cells to enable better study comparisons. These characteristics included that MSCs should be adherent to plastic when maintained in standard tissue culture conditions, be able to differentiate into osteoblasts, chondroblasts, and adipocytes, and also express or be deficient in a set of panel markers [32]. It has been suggested that all studies should show expression of CD73, CD105, and CD90 and no expression of CD45, CD34, CD14 or CD11b, CD79*α* or CD19, and human leukocyte antigen-DR (HLA-DR) to confirm that they are actually MSCs [32]. These are just a few of the conserved genes from the different MSC niches. Tsai et al. (2007) found that there were as many as 47 genes which were upregulated and 11 genes downregulated in their comparison of MSCs from amniotic fluid, cord blood, bone marrow, and amniotic membrane [33]. These conserved genes also had downstream effects on protein production where again it was observed that there are conserved cytokines and growth factors produced by MSCs such as macrophage migration inhibitory factor (MIF), interleukin-8 (IL-8), serpin E1, growth-regulated protein-*α* (GRO*α*), and interleukin-6 (IL-6), which are generally expressed by all MSCs from placenta, cord blood, and bone marrow [34]. These studies show that there are inherent similarities in all MSCs throughout the body that are conserved but at the same time there are subtle differences in gene expression and cellular function.

In the vast majority of MSC trials, very few studies actually characterised whether the cells collected had any MSC properties. Most were derived from the iliac crest and were then assumed to be bone marrow MSCs (BM-MSCs), but in most cases this was not substantiated. Some studies use the term bone marrow mononuclear cells (BM-MNCs) for the cells derived from the bone to make this distinction but overall this general lack of distinction does make comparisons between studies difficult. For example, Badiavas and Falanga (2003) isolated bone marrow from the iliac crest in patients using heparin syringes and within 2–5 minutes this aspirate was applied directly to the wounds and injected into the wound margins and then covered in several layers of dressings. The remaining aspirate was then cultured in modified Dexter cultures for up to 3 days at 33°C and 5% CO_2_ and readministered to the patients as required [35]. This method suggests that there would be a mixed population of cells used to treat the patient and none of which were actually probed for MSC markers. Mansilla et al. (2005) did not determine where the MSCs were harvested but do say that they were from a young healthy donor. These MSCs were not injected immediately but cultured in DMEM/10% foetal bovine serum. After 3 days the nonadherent cells were removed and the remaining cells cultured until confluent. This was repeated for 3 passages when the cells were then cryopreserved prior to use. When used these MSCs were introduced systemically via intravenous injection into the mouse tail vein [36]. The majority of these cells were reported to be CD44 positive, which again suggested a mixed population of cells. A third study by Arno et al. (2014) took the characterisation further by isolating Wharton's jelly-derived MSCs from umbilical cord and then culturing them in MSC culture medium CMRL (Gibco)/10% foetal bovine serum and 1% antibiotic/antimycotic solution. MSCs were then determined, by flow cytometry, by identifying the cells to be CD90^+^, CD73^+^, CD105^+^, CD45^−^, CD14^−^, CD34^−^, CD19^−^, and HLA-DR^−^. In this study MSC conditioned culture medium was used to treat the wounds rather than the MSCs themselves [37, 38]. These three independent studies show that there are a variety of techniques used to isolate and treat MSCs before their use. The first study used a mixed population of cells from the iliac crest without any characterisation, whereas Mansilla et al. reported on the MSCs adhering to tissue culture plastic and expressing CD44 but again there is a mixed population of cells used as not all cells were CD44^+^. Whereas these two studies used little characterization, Arno et al. cultured the cells and identified a significant array of cell markers to identify the MSCs; however unlike the previous two studies the MSC cultured medium was used instead of the MSCs themselves. This simple comparison shows the difficulties in comparing MSC treatments in skin repair.

Despite being able to isolate stem cells for over 4 decades and experience in treating wounds with MSCs for around 10 years now, there are actually relatively few clinical trials currently underway to investigate this. A search of ClinicalTrials.gov for “*mesenchymal stem cell* AND* wound*” revealed just 50 trials, 29 of which are open at the time of writing (October 2014). No doubt there are other trials that simply do not define their cells in a search-friendly way but our search provides an indication that there are still hurdles to be overcome.

As mentioned above Badiavas and Falanga (2003) were among the first to treat chronic wounds with MSCs. This resulted in increased cellularity of the wounds with the recruitment of inflammatory cells which were absent prior to treatment and the authors suggested that there were immature hematopoietic cells also present in the wound. There was an increase in reticulin fibres previously unseen in the wound and increased overall vascularity. All wounds which had previously failed to heal for more than 1 year completed the wound healing process after the application of MSCs [35]. Another of the earlier studies investigating the effects of human BM-MSCs on skin repair was conducted by Mansilla et al. (2005). Mice received a full-thickness skin defect dorsally and BM-MSCs were either injected systemically or applied to the wound in a polymer implant, which resulted in accelerated wound closure in the treated mice (in both cellular treatments) when compared to the vehicle controls [36]. Falanga et al. (2007) again used autologous MSCs harvested from patients with acute wounds from skin cancer surgery or from patients with chronic wounds. The MSCs were delivered within a fibrin spray and the cells were found to promote elastin production within the wound with a direct correlation between the reduction in wound size and the numbers of MSC applied [39]. A similar study, carried out by Dash et al. (2009), used autologous MSCs to treat 24 patients with nonhealing ulcers (diabetic ulcers and Buerger disease) of the lower limbs. Treatment with MSCs led to a reduction in ulcer size after 12 weeks with increased pain-free movement concurrent with an increase in fibroblasts and mature and immature immune cells found within the wounds [40]. Human BM-MSCs were also used by Stoff et al. (2009) to treat 3 cm long dorsal incisions in rabbits. Treated wounds had less granulation tissue and displayed a reduction in scarring along with an increase in tensile strength [41]. All of these studies together show that human MSCs could be used in a range of wound types to increase the rate of healing as well and reduce scarring and increase tensile strength. The evidence suggested that the effects seen here were largely due to improved dermal matrix deposition.

It is of course known that there are likely multiple modes of action and different models and situations will likely highlight these functional differences. Ichioka et al. (2005) isolated murine BM-MSCs to investigate their effects on angiogenesis in a wounding skin flap model. Wounds treated with BM-MSCs showed an increase in the density of functional capillaries when compared to controls on days 3, 5, and 7 after wounding. This led the group to test this application in a 48-year-old female who was suffering from a leg ulcer that was unhealed for over a year despite proper wound care and treatment. BM-MSCs were obtained from her iliac crest and then placed in a collagen matrix (TERDERMIS). This was placed in the ulcer for 2 weeks, following which there was an increase in vascularity in the wound, which was then fully healed with a split-thickness skin graft. There were no complications upon followup 1.5 years later [42]. Falanga et al. (2007) also found that BM-MSCs could promote angiogenesis. When treating wounds on the dorsum of diabetic mice with their MSC-fibrin spray they discovered that the BM-MSCs appeared to differentiate into vasculature to increase the rate of healing [39]. The immortalised human mesenchymal cell line V54/2 has also been shown to promote angiogenesis in excisional wound in nude mice by the production of VEGF and FGF-2 [43]. Lee et al. (2012) observed an improvement in vascularity in patients with critical limb ischemia whom they treated with intramuscular injections of adipose tissue MSCs (AT-MSCs). The treatment with AT-MSCs led to clinical improvements in 66.7% of patients together with a decrease in pain experiences and increased walking distances [44]. These studies show that MSCs are able to act favourably on native vasculature promoting angiogenesis not only via paracrine effects by the secretion of growth factors but also by directly differentiating into cells of the vasculature.

González Sarasúa et al. (2011) used BM-MNCs to treat pressure ulcers in patients suffering from pressure ulcers following spinal cord injury. These cells were obtained from the individual patients and applied to the pressure ulcers via injection during debridement and suturing of the wound. Full healing was observed in 19 of the 22 patients [45]. Lu et al. (2011) made a comparison of BM-MSCs, BM-MNCs, and saline to see which of the cell populations increased the rate of healing in diabetic ulcers. Cell suspensions of the different cell populations were made and then injected subcutaneously around the foot ulcers. Both of the cell treatments led to improved blood circulation around the wounds, as well as increase in the amount of pain-free time experienced by the patients. There were no amputations following the cell treatments (there were 6 in the saline treatment group) but the BM-MSCs show the fastest rate of healing when compared to the BM-MNCs and saline treatment [46]. Zebardast et al. (2010) harvested MSCs from human umbilical cord and then administered these cells in a fibrin delivery vehicle to dorsal excisional wounds in mice. The mice treated with MSCs showed a decrease in contraction of the wound, an increase in matrix deposition and wound strength, and a faster rate of reepithelialisation when compared to controls [47]. These investigations show that although BM-MSCs are the most frequently utilised there are other mixed cell populations and MSC niches that could be utilised successfully to treat differing wound types.

Most of the earlier MSC wound treatment research has focused on delivering MSCs to the wound site to enable them to differentiate into the required cells to promote the wound healing response. However, data from these studies suggest that the MSCs do not remain in the wound site for long periods or can even affect healing from distal sites [48]. More recently, along with improvements in protein separation, proteomics and mass spectrometry led to a focus on the factors released by MSCs and their paracrine effects [49–51]. This has, in some of the literature, been termed the secretome. These secreted factors come in a variety of guises including cytokines, chemokines, growth factors, ECM proteins/enzymes, and miRNA. Their effects may allow for increased cell proliferation, differentiation, and survival, as well as increased angiogenesis and a reduction in inflammatory responses [50, 52]. Overall, this can lead to improvements in tissue regeneration and the rates of healing. The majority of this research has come from probing MSC conditioned media in an attempt to identify the different elements of the secretome [50]. Furthermore this has led to encouraging results in the treatment of brain repair, cancer, lung and liver injury, kidney disease, and cardiovascular disease [53–58].

While the treatment of wounds with heterogeneous populations of BM-MNCs has shown promise, identifying the cells that have the most beneficial effect for a more defined therapy will deliver a more elegant and effective approach. A more patient/disease specific tailoring of MSCs treatment may be achieved by using the secretome of MSCs rather than direct MSC treatment. This is a promising area of discovery as it could lead to reduced biological variability and eliminate immunocompatibility, reducing the risk of tumorigenicity and the transmission of diseases [49, 59].

## 4. Hematopoietic Stem Cells

Hematopoietic stem cells (HSCs) are, as the name suggests, a blood cell progenitor cell that give rise to all the blood cells. There is little literature discussing the use of purified HSCs as a wound repair strategy but therapies that utilise bone marrow aspirates no doubt contain a mixture of MSCs, HSCs, and a range of other multipotent progenitor cells. It is understood that HSCs are mobilised towards the site of cutaneous wound repair and play an important role in the regulation of the proliferation and migration of epithelial and dermal MSCs [60].

Most HSCs express CD45, and a subset of HSCs also express CD34. These markers can be used for identification, as MSCs do not express either one [61]. The CD34^+^ HSC is the most abundant cell type in the bone marrow and can be harvested from either bone marrow or peripheral blood. Isolation from peripheral blood is the most common method as HSCs can be mobilised into circulation by the administration of cytokines such as granulocyte macrophage colony stimulating factor (GM-CSF) [62]. It should be noted however that if a single marker such as CD43 is used for purification then there will likely be other non-MSC contaminants such as EPCs.

There is little discussion in the literature of hematopoietic therapies. This is most likely due to the lack of robust identification and isolation methods. The use of CD34^+^ cells is, however, described in the literature. It can be postulated that this isolated population will consist largely of HSCs. It has been shown that bone marrow derived cells from the hematopoietic lineage increase in number in cutaneous wounds and lead to wound contraction [61]. This makes them a candidate for therapeutic investigation.

A recent clinical trial conducted by Wettstein et al. (2014) investigated the effect of using autologous hematopoietic stem cells in chronic wounds in a pressure sore model [63]. Three patients underwent cell harvest from the iliac crest and the CD34^+^ cells were isolated and injected as a cell suspension into the wounds. There was a decrease in wound size on the side treated with cells although this was not shown to be statistically significant [64]. The outcome that was highlighted is that of safety; a two-year followup indicated no signs of malignancy. Although this study was small in size and the results were not ground-breaking, it identifies an important model for further study of cell therapies in humans.

## 5. Endothelial Progenitor Cells

Endothelial progenitor cells (EPCs) can be isolated from peripheral blood or bone marrow. There are a wide range of surface markers that can be used to characterise EPCs; however they are generally agreed to be CD34^+^, CD133^+^, and VEGFR-2^+^ [65, 66]. They are a heterogeneous population of mononuclear cells that have been selected for their enhanced potential to differentiate into endothelial cells [66]. EPCs have been shown to migrate towards wounds, tumours, and areas of reduced blood supply, ischemia [67]. Whilst the vast majority of research into EPCs focuses on the use of EPC therapy for treatment of stroke [68–70] and other vascular diseases there is recent research into the possible use of EPCs for cutaneous wound healing.

Leg and foot ulcers are often accompanied by a compromised vasculature. Therefore proangiogenic EPCs are a logical therapeutic target. EPC therapy has been shown to increase healing in cutaneous wounds by enhancing the neovascularisation of granulation tissue* in vivo* [71]. EPCs are recruited to ischemic regions from the bone marrow and incorporated into the growing vasculature, summarised in [72]. A number of mobilisation triggers have been identified and these include hypoxic tissue gradients, endothelial nitric oxide synthase, nitric oxide, VEGF-A, MMP-9, and GM-CSF [67, 73]. One of the proposed mechanisms for the success of hyperbaric oxygen and negative pressure treatment of ischemic wounds is the mobilisation of EPCs and other stem cells [67, 74, 75], as well as the release of angiogenic factors such as VEGF, PDGF, macrophage inflammatory protein (MIP), and FGF-2 [73, 76–78].

There is evidence to suggest that EPCs are defective in patients suffering from diabetes [79–82]. These EPCs demonstrate impaired mobilisation and homing, decreased proliferative potential, and increased rates of apoptosis which results in reduced EPC numbers and delayed wound healing [83]. Therefore the application of EPCs directly into these compromised wound beds is an attractive therapeutic option. Various delivery vehicles have been investigated for the delivery of EPCs. Kim et al. (2009) describe the use of a biodegradable poly(L-lactide) scaffold grafted with RGD to deliver EPCs to a murine wound model. This study also showed that delivery of cells in this fashion resulted in improved cell survival over the control injection [84].

## 6. Multipotent Progenitor Cells

Although the terms stem cell and progenitor cell are often equated, there is a growing consensus that progenitor cells are different. Progenitor cells are thought to be closer to a differentiated target cell than a stem cell. The most important difference is that while stem cells can divide an unlimited number of times, progenitor cells cannot. Here lies the safety advantage of the progenitor cell as a therapy; the progenitor cell has a finite life whereas the stem cell is a potential risk for the development of malignancy. As with MSCs, the most common sources of multipotent progenitor cells (MPCs) are either bone marrow or adipose tissue. There is some evidence to suggest that MPCs sourced from adipose may have a more immunomodulatory effect [85].

A type of MPCs, known as multipotent adult progenitor cells (MAPCs), was first identified over a decade ago with characteristics similar to most adult somatic stem cells; they proliferate without senescence and have a broad differentiation ability [86, 87]. It was demonstrated that they could be expanded* in vitro *for greater than 70 population doublings, far more than equivalent human MSCs (20–25 doublings) [88]. A recent study has verified that hMAPCs and hMSCs are two distinct cell populations, in contrast to hMSCs, hMAPCs are negative for CD140a, CD140b and alkaline phosphatase (ALP) but express low levels of major histocompatibility complex class I (MHC class I) [89, 90].

A particularly promising aspect of human derived MAPCs (hMAPCs) is their immunomodulatory capability; they have been shown to exert strong immunosuppressive effects on T-cell proliferation [89]. Although the mechanism of this effect is not fully understood, the results correlate with hMSCs. This was the first time this effect was shown in hMAPCs paving the way for further clinical trials. Although these immunosuppressive capabilities remain largely unexplored for wound repair and the desirability for immunosuppression within a wound site remains unclear, there is much interest in the utilisation of hMAPCs for the treatment of graft-versus-host disease (GVHD), specifically, for clinical trials exploring the potential of hMAPCs to prevent the rejection of transplanted hematopoietic stem cells and enhance engraftment in cases of leukaemia and reduce organ rejection after a liver transplantation [91, 92].

A leading commonality of cutaneous wounds is ischemia and localised hypoxia; this leads to a chronic wound microenvironment in which cells are senescent or phenotypically altered [93, 94]. It was shown that when hMAPCs were applied in a murine model of moderate limb ischemia, angiogenesis was significantly increased. It was also shown that endogenous stem cell proliferation was increased leading to reduced ischemia and improved function [95]. The clinical potential of these cells is obvious and the real key is their high proliferative capacity. This allows for a well-characterised single donor product to be used in large clinical trials. In the very near future we should expect to see hMAPCs applied in cutaneous wound repair in human clinical trials.

While the bone marrow often seems to be the main source of therapeutic cells, the skin itself may hold the potential for cutaneous repair. There is mounting evidence to suggest that the skin may be a source of MPCs [96, 97]. These cells have the advantage that they are more easily accessible than those from bone marrow and adipose. Moreover, skin-derived MPCs originating from the hair follicle are present in an immune privileged environment which may allow allogeneic use of these therapeutic cells with a reduced risk of rejection [98].

## 7. Cell Delivery

The delivery of cells to cutaneous wounds presents a unique and specific set of challenges. Although the injection of a suspension of cells may be the simplest method, this may not be the optimum method to deliver cells to a cutaneous wound. First of all, direct injection into the wound may be difficult in long standing leg ulcers that have a thick layer of dense scar tissue. To bypass this issue and to also provide a more receptive environment for administered cells, debridement of the wound site is often performed before the delivery of cells. The debridement removes any scar tissue, fibrin, and necrotic tissue and also achieves bleeding from the wound bed. This bleeding aims to tackle the second issue; most chronic wounds are ischemic in nature. They exhibit poor blood flow and present a hostile environment for any cells. It is theorised that, by inducing bleeding, this may be enough to mitigate the hostile environment for long enough for the administered cells to take hold and stimulate a healing cascade.

The published therapeutic trials outlined in Table 1 all deliver adherent cells in a suspension or hydrogel matrix. While this has become almost standard procedure for cell therapies, the aforementioned limitations may lead to the future development of alternative approaches with the goal of reduced costs or increased efficacy [99].

Yoshikawa et al. (2008) tested the effects of human BM-MSCs in a murine wound healing model before progressing to small clinical trials in humans with chronic wounds. In the initial mouse experiments human BM-MSCs either were implanted onto a collagen sponge which was then placed adjacent to a dorsal wound in the nude mouse or were injected intradermally around the wound. These studies were compared to saline controls and the data illustrated that only the BM-MSC implanted collagen sponges showed any improvement in repair with increased vascularity, fat, and matrix deposition compared to the other groups. This method was then used in 20 patients whose chronic wounds were treated with collagen sponges containing autologous BM-MSCs. Eighteen of the twenty patients showed some improvement when treated with the MSCs and the data showed the treatment to be effective [100]. This study was the first to suggest that the use of scaffold for administering the MSCs to the wound may be more beneficial than just injecting the MSC around the wound site. The delivery of cells is an important and often neglected aspect of any therapeutic approach. As cell therapies mature, we will no doubt see new and innovative delivery approaches to try and maximise efficacy.

## 8. Conclusions and Clinical Directions

The ability to utilise an allogeneic population of cells in a cell therapy will always make better business sense and certain derived progenitor cells have been shown to be immune privileged [89]. These therapies have shown great potential, scientifically, but this is just one side of the coin. Commercial use of allogeneic cells is more complex and requires additional regulatory, legal, and safety hurdles to be overcome. The coming decade is sure to see both autologous and allogeneic business models for cell therapy.

Cell therapies have been subject to massive hype and media coverage over the last decade and while this may well be the century of the cell, there are concerns that the clinical use of cell therapies is beginning to outpace the evidence. While animal studies and small scale clinical studies support the potential benefit for cell therapies in wound repair, clinical barriers include a lack of sufficient clinical evidence, high costs, and a lack of standardised delivery techniques [83]. The future use of more standardised methods would allow for better comparisons; this will help build a more robust pool of clinical evidence, essential for stem cell therapies to be taken forward.

Mechanisms of action are broadly understood but they need to be further defined and models need to be developed to better evaluate modes of action. While the treatment of wounds with heterogeneous populations of cells has shown promise, identifying the cells that have the most beneficial effect for a more defined therapy will deliver a more elegant and effective approach. It makes business sense to minimise risk and this can be achieved with a better defined final product.

## Figures and Tables

**Table 1:** Published clinical use of cell therapies in human cutaneous wounds.

Wound type	Cell type	Delivery system	Outcome	References
Pressure sores	Bone marrow CD34^+^ (HSCs)	Injected locally	Validation of test model but no significant enhancement over standard (noncell) treatment methods.	[64]

Type 2 diabetic limb ischemia	Bone marrow MSCs OR bone marrow mononuclear cells	Injected intramuscularly	No adverse reactions to cell injections. BM-MSCs lead to improved healing rate at 6 weeks and reached 100% 4 weeks earlier than BM-MNCs. No difference with respect to pain relief and amputation.	[46]

Type IV pressure ulcers	Bone marrow mononuclear cells	Injected locally	Mean intrahospital stay was reduced from 85 to 43 days. At a 19-month followup, none of the ulcers had recurred.	[45]

Nonhealing ulcers	Adipose MSCs	Injected intramuscularly	Clinical improvement in 67.7% of patients. At 6 months, improved pain rating and walking distance.	[44]

Nonhealing ulcers	Bone marrow MSCs	Injected locally	Improvement in pain-free walking distance and reduction in ulcer size.	[40]

Intractable dermatopathies	Bone marrow MSCs	Collagen sponge	18/20 wounds healed (2/20 patients died).	[100]

Acute wound (following skin cancer resection). Chronic wound (foot or leg, greater than 1 year old).	Bone marrow MSCs	Fibrin spray	Correlation with number of cells applied and reduction of chronic wound size.	[39]

Radiolesions	Adipose MSCs	Injected locally	Improvement or remission of symptoms in all patients.	[101]

Radiation burn	Bone marrow MSCs	Injected locally	Reduction in pain leading to complete healing (single patient).	[102]

Chronic wounds	Bone marrow derived cells	Injected and applied directly to wound	Enhanced clinical response.	[103]

Diabetic ulcer	Bone marrow suspension	Collagen matrix	Generation of vascularised tissue able to accept skin graft (single patient).	[42]

Chronic wounds	Bone marrow derived cells	Topical application of cell suspension	Complete wound closure in all three patients.	[35]

## Abbreviations

AT-MSC: Adipose tissue mesenchymal stem cell
ALP: Alkaline phosphatase
BM-MSC: Bone marrow mesenchymal stem cell
BM-MNC: Bone marrow mononuclear cell
CD: Cluster of differentiation
EPC: Endothelial progenitor cell
FGF-2: Fibroblast growth factor-2
GVHD: Graft-versus-host disease
GM-CSF: Granulocyte macrophage colony stimulating factor
GRO*α*: Growth-regulated protein-*α*
HSC: Hematopoietic stem cell
HLA-DR: Human leukocyte antigen-DR
IL-[number]: Interleukin-[number]
MIP: Macrophage inflammatory protein
MIF: Macrophage migration inhibitory factor
MHC class I: Major histocompatibility complex class I
MMP: Matrix metalloproteinase
MSC: Mesenchymal stem cell
MAPC: Multipotent adult progenitor cell
MPC: Multipotent progenitor cell
PDGF: Platelet derived growth factor
TIMP: Tissue inhibitor of metalloproteinase
TLR: Toll-like receptor
TGF-*β*: Transforming growth factor-*β*
VEGF: Vascular endothelial growth factor.
